# Melatonin Regulates Growth‐Arrest and DNA‐Damage Genes GADD153, GADD45g and GADD34 in Experimental Varicocele

**DOI:** 10.1002/cbf.70308

**Published:** 2026-09-11

**Authors:** Esra Önal, Numan Baydilli, Derya Karabulut

**Affiliations:** ^1^ Health Sciences Institute Erciyes University Kayseri Türkiye; ^2^ Department of Histology‐Embryology, Faculty of Medicine Erciyes University Kayseri Türkiye; ^3^ Department of Urology, Faculty of Medicine Erciyes University Kayseri Türkiye

**Keywords:** GADD, GDNF, hormones, melatonin, StAR, Varicocele

## Abstract

This study aimed to reveal the relationship between experimental varicocele and melatonin levels. Forty adult Wistar albino rats were used in this study. Control (C), Melatonin (M), Varicocele (V), Varicocele+Melatonin (V + M) were planned. Varicocele was created on the first day of the experiment by opening the abdominal area, narrowing the left renal vein with a 0.85 mm metal wire and using a 4‐0 silk suture, after removing the metal wire, the animal was closed (with a 3‐0 silk suture) and kept for 60 days. Melatonin was administered at 10 mg/kg ip (intraperitoneal) on the 61st day, every 24 h for 7 days. Histological analysis and GADD34, GADD45g, GADD153 immunohistochemical staining were performed. Additionally, StAR and GDNF gene expressions were examined. ABP, FSH, LH, Testosterone, Melatonin hormones, TAS and TOS levels were evaluated from blood serum taken from experimental animals. According to our results, group V showed a decrease in testicular weights, seminiferous tubule diameter, and JTBS score, while GADD34, GADD45g and GADD153 proteins increased. In group V, testosterone and TAS levels decreased and TOS levels increased. In group V, testosterone, FSH, ABP, melatonin levels decreased and LH level increased. StAR and GDNF gene expression also decreased in group V. In the V + M group where melatonin was applied, an improvement was observed in biochemical parameters as well as histological, immunohistochemical and gene expression analyses. During varicocele, endogenous melatonin level decreases and activates various mechanisms and hormones in the tissue, including GADDs, causing loss of structure and function, while melatonin acts as a good therapeutic on varicocele.

## Highlights

- Growth‐arrest and DNA‐damage genes GADD153, GADD45g, and GADD34 are affected in experimental varicocele.
- Melatonin can regulate GADD153, GADD45g, GADD34, and StAR.
- Melatonin may be an important therapeutic target in varicocele.

## 1 Introduction

Varicocele, a common problem in reproductive medicine, occurs as a result of abnormal venous dilation and/or tortuosity of the pampiniform plexus in the scrotum [1]. While approximately one‐third of infertile men present to the clinic with varicocele, its prevalence in the general population is approximately 15% [2]. Varicocele is also considered the most common correctable cause of male infertility. Clinical varicocele diagnosis is made by physical examination and open surgery; laparoscopic and percutaneous techniques are used as correction methods [3]. Although varicocele is considered a reversible cause of male factor infertility, it continues to be the subject of research on increasing treatment options due to its negative effects on the male reproductive system and the risk of recurrence.

Studies conducted to date have shown that there is a relationship between varicocele and decreased male fertility potential. The primary pathophysiological mechanisms of varicocele are scrotal hyperthermia and increased scrotal temperature, which cause testicular endocrine dysfunction, spermatogenesis, and testicular dysfunction [1]. It also causes low serum testosterone levels due to impaired Leydig cell function [4]. Multiple factors are effective in the mechanisms of varicocele that may cause infertility. Therefore, it is accepted that the pathogenesis of varicocele is quite complex and multifactorial. Another important factor in this complex pathophysiological process is oxidative stress. Venous reflux occurring after varicocele causes an increase in hydrostatic pressure, which in turn causes an increase in scrotal temperature, hypoxia and the accumulation of toxic metabolites. Thus, increased oxidative stress in the tissue leads to increased reactive oxygen species and decreased total antioxidant capacity [5]. These effects affect both spermatogenic and non‐spermatogenic cell populations, leading to testicular dysfunction. Depending on the severity of varicocele, semen quality gradually decreases [6]. Therefore, among people suffering from varicocele, the variability in fertility from individual to individual is directly related to the severity of the varicocele. Our aim here is to make antioxidant defense systems more sensitive to reactive oxygen species damage by imitating the infertility problem with the experimental varicocele model. It has been reported that melatonin production is secreted very little or not at all before the age of 3 months in the first years of life, production starts later and reaches its highest level between the ages of 1‐3, and when it reaches adult levels, the night peak level gradually decreases by 80% [7]. The decrease in blood melatonin levels as age progresses may contribute to the development of diseases such as varicocele.

With the hydrostatic pressure and hyperthermia that occur after a varicocele, the balance between oxidant/antioxidant in the tissue is disrupted, and oxidative stress occurs. This oxidative stress triggers various mechanisms, especially endoplasmic reticulum (ER)‐stress. Thus, cellular homeostasis is disrupted, and the cell's function and structural integrity deteriorate, leading to cell death. In this study, for the relationship between varicocele/ER‐stress, growth arrest and DNA damage (GADD)‐inducible gene (GADD153) in spermatogenic cell lines, GADD45g and GADD34 induced by genotoxic stress and growth arrest signals were immunohistochemically evaluated, ER‐stress‐induced apoptosis, in addition to revealing the intrinsic factors in the etiology of varicocele by demonstrating non‐spermatogenic cell function via glial cell‐derived neurotrophic factor (GDNF) and steroidogenic acute regulatory protein (StAR, and examining supportive hormone parameters and melatonin levels, it was also revealed whether the molecules mentioned above are involved in the pathogenesis of varicocele.

## 2 Materials and Methods

### 2.1 Experimental Design

This project was carried out with male adult Wistar albino rats, approximately 10 weeks old and weighing 200–300 gr raised at Erciyes University Experimental and Clinical Research Center (DEKAM). During this process, rooms were automatically air‐conditioned with a 12‐h light/dark cycle and a constant temperature of 19°C–21°C. The rats were fed with classic pellet food and water. Approval was received for this study from the Erciyes University Animal Experiments Local Ethics Committee (date 08.09.2021, meeting number 08, decision number 21/185).

**Figure 1:**
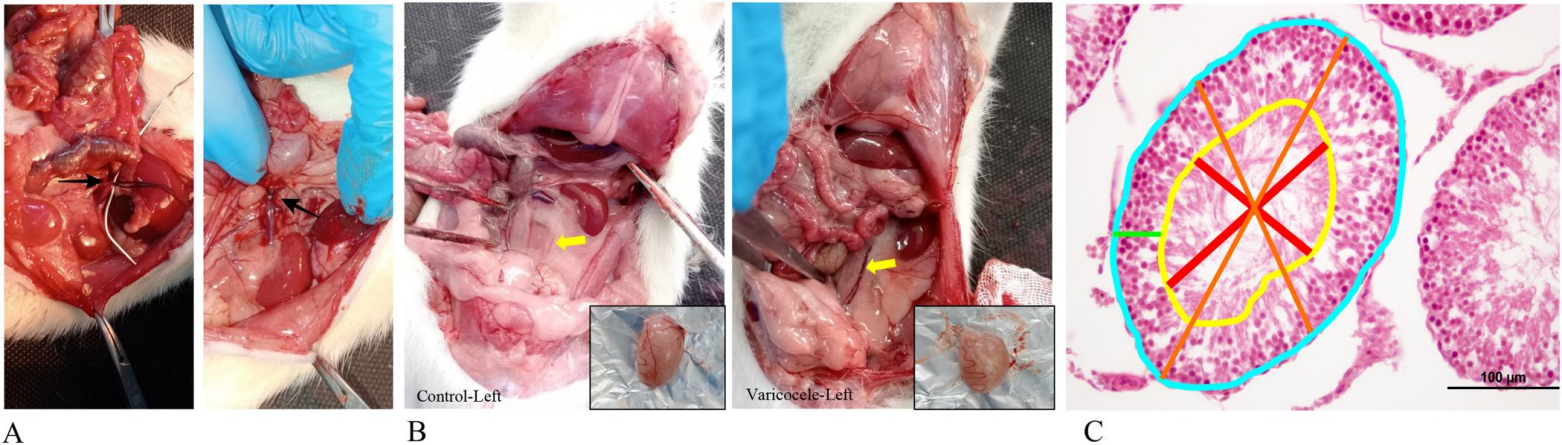
Experimental design. (A) Narrowing of the left renal vein with metal wire and suture (Black arrow), On the right is the final state of the left renal vein tied with suture at the end of the procedure. (B) At the end of the experiment, the left internal spermatic vein (yellow arrow) enlarged after the left renal vein ligation of groups C and V and the testicular samples of these animals are shown in the small picture. (C) The diameter of the seminiferous tubule marked in blue was measured as indicated by the orange lines. The seminiferous tubule lumen marked in yellow was measured as indicated by the red lines. The seminiferous epithelium thickness was measured as indicated by the green line.

Experimental groups of the study; Control group (C) (*n* = 10): was given general anesthesia, and after reaching the abdomen with a surgical procedure, the left renal vein was exposed and the anterior abdominal wall and skin were closed with a full‐thickness 3‐0 silk suture and the procedure was terminated. No solvent was applied to the control group (C). Melatonin group (M) (*n* = 10): 10 mg/kg melatonin was administered intraperitoneally (ip) to the animals for 7 days and the animals were killed on the 8th day. Rats in this group were slaughtered in a way that coincided with the slaughter of animals in other groups. Melatonin dosage was determined regarding literary sources [8]. Melatonin was prepared freshly at the same time every day by dissolving it in ethanol 96% (500 μL), taking into account the weight of the animals. Then, it was diluted with physiological saline and injected into the animals. Thus, the final concentration of ethanol was kept below 5% (Derya [9]. Varicocele group (V) (*n* = 10): under general anesthesia (xylasin 5–10 mg/kg, ketamine 50–70 mg/kg, ip), a 4.0 silk suture was placed in the upper left abdominal region, which would be opened from the midline, and the section where the renal vein ligation would be performed. The narrowing process was performed on a 0.85 mm diameter metal wire and the wire was pulled after the vein was narrowed. With this procedure, the outer diameter of the vessel was reduced to 1 mm [10, 11]. The abdominal wall and anterior abdominal muscles were then closed. They were kept for 2 months (60 days) and the animals were killed on the 61st day. The process performed is shown in Figure 1A. Varicocele+Melatonin group (V + M) (*n* = 10): It was accepted that varicocele was formed 60 days after creating varicocele by applying the varicocele creation procedures mentioned above. Starting from the 61st day, 10 mg/kg melatonin was administered ip for 7 days and the animals were killed on the 68th day.

At the end of the experiment, the rats were weighed and their weights recorded. They were anesthetized with ketamine + xylazine. Blood samples were then taken from the abdominal vena cava. A sperm sample was taken from the left epididymis tissue exposed by opening the scrotal sac, and sperm analysis was performed. The left testicular tissue was quickly removed, weighed, and recorded. The left testicular tissue was divided into two halves; half was used for histological procedures, and the other half was divided again, with one half placed in trizol tubes for gene expression analysis and the other half stored at ‐80°C for oxidant/antioxidant status determination. Images showing the successful application of the experimental model are shown in Figure 1B.

### 2.2 Spermiogram (Semen Analysis)

To evaluate the sperm count in terms of number, the sperm sample was diluted with 1 ml of spermwash from the cauda part of the ductus epididymis of the rats and pipetted, and then the sperm sample diluted with spermwash was dropped onto the machine. Counting was done under the microscope with a 20‐gauge objective after the camera's cover glass was closed. For semen analysis, motility and count were evaluated [12].

### 2.3 Histopathological Analyzes

Testicular tissues were placed in a 4% formaldehyde solution. They were then quickly washed with tap water. First, they were immersed in increasing amounts of alcohol solutions (50%, 70%, 80%, 96%, 3 × 100%). Then, they were immersed in xylene solution three times. Testicular tissues, after being immersed in liquid paraffin, were fixed by embedding them in paraffin. 5 μM‐thick sections were cut from the tissue blocks. Hematoxylin‐eosin (H&E) staining was performed. In the sections, the Johnsen Testicular Biopsy Score (JTBS) was measured, as well as the seminiferous tubule diameter, seminiferous epithelial thickness, and seminiferous tubule lumen width (D [13]). All measurements were made as 10 animals × 20 tubule measurements from each group, and statistics were made as 150 tubules. JTBS score [14] 1: neither germ cells nor Sertoli cells present, 2: no germ cells, 3: only spermatogonia present, 4: only a few spermatocytes present, 5: no spermatozoa or spermatids but many spermatocytes present, 6: only a few spermatids present, 7: no spermatozoa but many spermatids present, 8: only a few spermatozoa present, 9: many spermatozoa present but irregular spermatogenesis, 10: complete spermatogenesis and perfect tubules.

Seminiferous tubule diameter, seminiferous epithelial thickness, seminiferous tubule lumen width were obtained from images taken under an Olympus BX51 microscope with a 40x objective [15]. The illustration of how the measurements took place was shown in Figure 1C. To determine the histopathological changes occurring in the testicular tissue and the differences between the groups, the amount of edema, number of eosinophilic tubules and atrophic tubules in randomly selected areas, interstitial connective tissue and seminiferous tubules were scored in each preparation of the groups. This scoring was carried out semi‐quantitatively, giving a value between 0 and 3. According to the scoring: 0: none, 1: little, 2: moderate, 3: severe.

### 2.4 Immunohistochemistry Analysis

Expression of GADD34, GADD45g and GADD153 was determined in left testicular tissues. Testicular tissue sections were kept at 60°C overnight and kept in xylene and alcohol. Phosphate buffer (PBS) was used for washing between treatments. Citrate buffer was used for antigen retrieval. A staining kit (TA‐125‐HDX, ThermoScientific) was used for the procedures after applying hydrogen peroxide (H_2_O_2_). GADD34 (Bioss, USA), GADD45g (Bioss, USA), GADD153 (Bioss, USA), GDNF (Bioss, USA) and StAR (Elabscience, USA) antibodies were used as primary antibodies. Diaminobenzidine was used to make immunoreactivities visible. Sections counterstained with Gill hematoxylin were washed several times with distilled water. The sections were passed through alcohol and xylene, respectively, and closed with sealing solution (Entellan, Merck). After light microscopic photographs were taken of the testicular tissues stained for immunoreactivity, measurements were made in the Image‐J program and evaluated statistically [16]. StAR immunoreactivity was measured from the interstitial area where Leydig cells are located, while all other proteins were measured in the seminiferous tubules.

### 2.5 Apoptosis

To demonstrate apoptotic cells, the Roche In Situ Cell Detection Apoptosis Fluorescein Kit was used. The staining kit was applied after paraffin removal from the tissue. All procedures were performed according to the kit's instructions. Tissues were counterstained with 4’,6‐diamidino‐2‐phenylindole (DAPI), and tissues coated with glycerol capping solution were imaged and photographed on an Olympus BX‐51 fluorescence microscope at a wavelength of 450‐500 nm. TUNEL‐positive cells were counted in ten randomly selected areas from each preparation, both in connective tissue and seminiferous tubules.

### 2.6 Gene Expression Analysis

Real‐time PCR analyses were performed to determine mRNA expression levels for GDNF and STAR genes in left testicular tissue. Genes 5‐sequences‐3: GDNF‐rno‐F; CCAGTGACTCCAATATGC, GDNF‐rno‐R; TTGATGGTGGCTTGAATA, StAR‐rno‐F; TCTCTACTTGGTTCTCAA, StAR‐rno‐R; TTACTTAGCACTTCATCTC, ACTB‐rno‐F; TACACTGATCCAGAAGAG, ACTB‐rno‐R; AGTAGTCCGAAGAATGAA. This entire analysis was carried out by DİAGEN Biotechnology company by purchasing services. TRIzol Reagent was used to ensure high quality Total RNA extraction. Nucleic acid loads (ng values) of the samples obtained from the total RNA extraction process were fixed to a certain ng value to be used in the next steps of the study. Reverse Transcriptase process was performed with Oligo (dT) and Random Hexamer primers to detect pre‐mRNA and mature‐mRNA structures. The cDNA obtained by synthesizing the opposite copy of the mRNA sequence with the Reverse Transcriptase enzyme was used as a template for standard PCR. RT‐qPCR procedures were performed on the Biorad CFX96 (Biorad, USA) device. Within the scope of the study, SensiFAST SYBR No‐ROX Kit (Bioline, UK) was used to determine gene expression levels. Setups were performed at the appropriate Tm value for each gene region and cycle curves and Melting Curve data were examined. cDNA chains created with mRNA sequences were used in relative expression analyses. For each of the samples in the experimental groups, the sequences of the relevant mRNA and its corresponding reference gene were amplified using a real‐time PCR device. As a result of the process, the data obtained from the device was evaluated with the 2^‐(ΔΔCt)^method and the threshold value (Ct, Cp, Cq) taken from the point where the fluorescence value exceeded the threshold value (crossed the Threshold line) in the studies carried out over 40 cycles was used in the calculations.

### 2.7 Biochemical Analysis

At the end of the experiment, blood samples taken from the abdominal vena cava of anesthetized animals were centrifuged at 10,000 rpm for 15 min. After centrifugation, blood serum samples were aliquoted into eppendorf tubes and stored at −80°C for further biochemical analysis. Melatonin, Testosterone, LH and FSH levels were determined by ELISA method in blood serum samples obtained from experimental animals.

A piece of testicular tissue taken from the animal's left testicle was stored at ‐80°C. Then, after being homogenized in a homogenizer under ice, it was centrifuged at 10,000 rpm at +4°C for 20 min, and then the upper part was transferred to another eppendorf tube, the pellet part was discarded and stored at ‐80°C. Melatonin and ABP levels, as well as total oxidant status (TOS) and total antioxidant status (TAS) were determined by the ELISA method from the left testicle homogenate taken from experimental animals.

### 2.8 Statistical Analysis

All statistical analyzes carried out in this project were carried out using GraphadPrism 7 program. Whether all data showed normal distribution was determined by the D'Agustino & Pearson normality test. Analysis of normally distributed data was carried out with One‐Way ANOVA. Tukey's multiple comparison test was used to determine differences between groups. The Kruskal‐Wallis test was used to analyze data that did not show normal distribution. Dunn's multiple comparison test was used to determine the statistical differences between groups in these data. *p* < 0.05 was considered significant.

## 3 Results

### 3.1 Determination of Animal Weights and Testicle Weights

No differences between groups were observed in the statistical analysis of the weights recorded at the beginning of the experiment. At the end of the experiment, it was determined that there was an increase in the weight of the animals. It was determined that the testicular weights of the groups were not distributed normally and the Kruskal‐Wallis test was applied. According to our results, it was determined that there was no statistically significant difference between the groups in left testicular weight. The weights of the animals weighed at the beginning and end of the experiment and the statistical results of the left testicle weight are shown in Table 1.

**Table 1:** Animal weights at the beginning and end of the experiment and measurement results of left testicular weight of the experimental groups.

	C	M	V	V + M	*p*
Animal weights (gr)	338.1 ± 25.63	340.4 ± 45.39	347.4 ± 30.36	342.9 ± 18.07	0.9225
Left testis weight (gr)	1.40 (1.37–1.57)	1.42 (0.92–1.61)	1.44 (0.58–1.52)	1.47 (0.92–1.54)	0.9537

*Note:* Animal weights were expressed as mean ± standard deviation, testicular weights as median (minimum‐maximum). There is no statistical difference between the groups. *p* < *0.05* was accepted as significant.

Abbreviations: C; control, M; melatonin, V; varicocele, V + M; varicocele + melatonin.

### 3.2 Spermiogram Findings

At the end of the experiment, sperm counts of all groups were performed and statistical analysis was performed. A statistically significant decrease was observed in the number of sperm in group V compared to groups C and M. It was determined that it increased slightly in the V + M group compared to the V group. Statistical differences between groups are shown in Table 2. Sperm motility, including in situ motility and forward motility, was observed in groups C and M. While it was observed that there were mostly immotile and forward motile sperms in the V group, the number of immotile sperms was observed to be less in the V + M group, and the number of motile and forward motile sperms was higher.

**Table 2:** Spermiogram analysis results of the experimental groups. The given sperm numbers were calculated as *10^6^.

	**C**	**M**	**V**	**V** + **M**	***p***
Spermiogram	59 ± 73.2^a^	59.5 ± 36.09^a^	10 ± 12.2^b^	14.1 ± 8.5^ab^	0.0081

*Note:* Data are expressed as mean ± standard deviation. No statistical difference was found between groups expressing the same letter (a, b). *p* < *0.05* was accepted as significant.

Abbreviations: C; control, M; melatonin, V; varicocele, V + M; varicocele + melatonin.

### 3.3 Histopathological Findings

**Figure 2:**
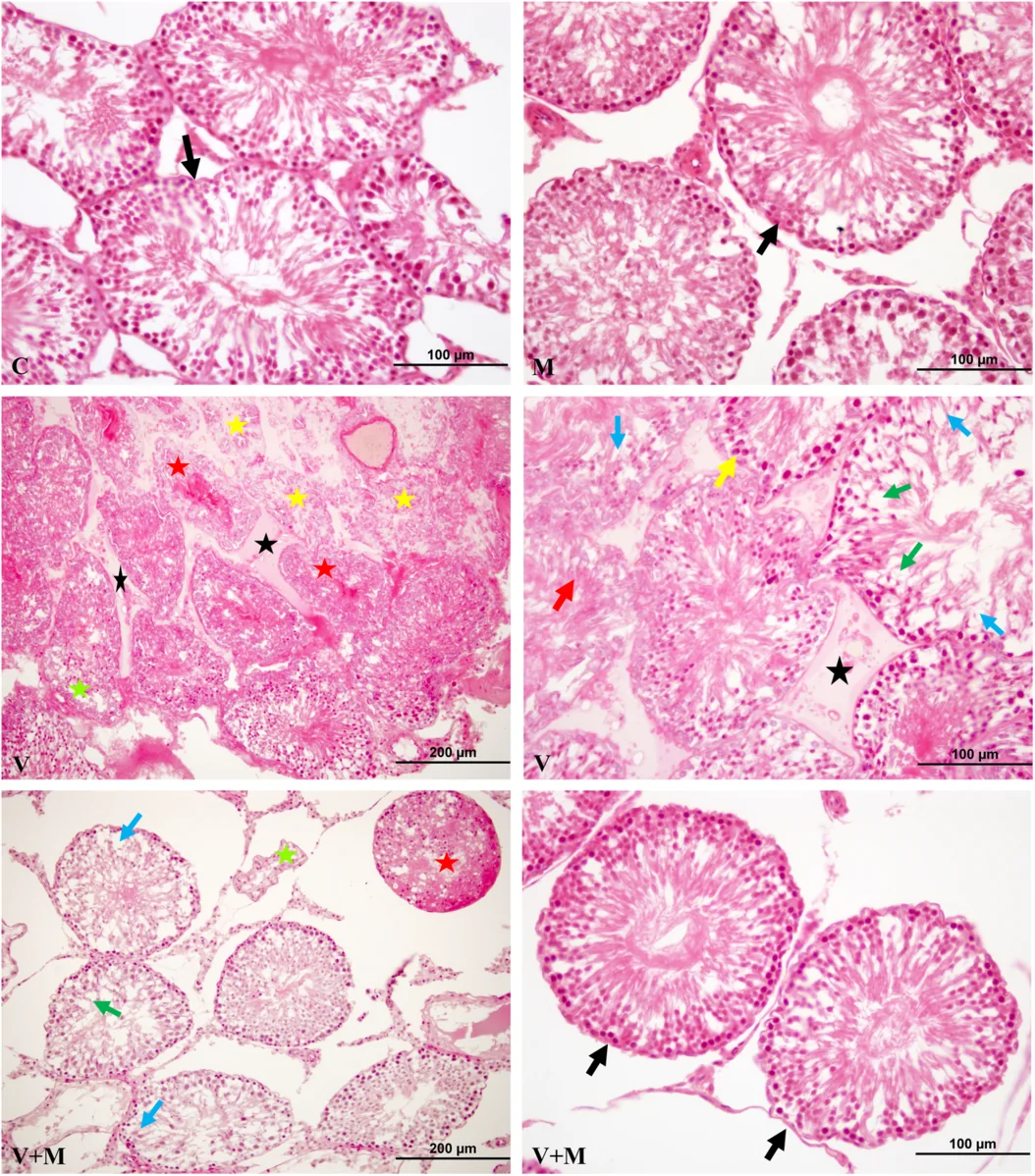
H‐E stains of experimental groups. Testicular tissue sections of groups C and M have regular histological appearance in terms of seminiferous tubule structure (black arrow) and interstitial connective tissue. In group V testicular tissue sections, irregular intertubular connective tissue as well as interstitial edema (black star), edematous tubule (yellow star), eosinophilic tubule (red star), atrophic tubule (green star) are shown. In group V, healthy seminiferous epithelium (yellow arrow), irregularly damaged seminiferous epithelium (red arrow), shed seminiferous epithelium (blue arrow), and vacuolization‐like spaces in seminiferous tubules (green arrow) are shown. In group V + M testicular tissue sections, both seminiferous tubule structures distinguished by their normal histological structure (black arrow) are observed, and eosinophilic tubule (red star), and atrophic tubule (green star) structures are shown. In addition, some tubules in this group showed shed seminiferous epithelium (blue arrow) and vacuolization‐like spaces in seminiferous tubules (green arrow). Images are shown as 20 × objective (scale bar: 200 µM), and 40 × objective (scale bar: 100 µM). C; control, M; melatonin, V; varicocele, V + M; varicocele+melatonin.

In the C and M group sections, the seminiferous tubule structure was regular in terms of both seminiferous epithelium and intertubular connective tissue, and spermatozoa were distinguished in the center of the seminiferous tubules. In group V, it was observed that there were wide gaps between the seminiferous epithelium of some tubules, the seminiferous epithelium was shed in most tubules, and the connective tissue between the tubules was irregular. In addition, necrotic tubules, distinguished by dark eosinophilic staining, were also observed in tissue sections, while edematous atrophied tubules without seminiferous epithelium were observed. While the presence of edema was often noted among the seminiferous tubules, both healthy seminiferous epithelium and seminiferous epithelial tubules with edematous, light‐colored nuclei were found in the tubules around the edematous areas. In the V + M group, it was observed that the seminiferous tubule epithelial structure was mostly preserved and the intertubular connective tissue exhibited a more regular structure. In the sections of this group, regular tubules with preserved seminiferous epithelial structure were frequently observed, while necrotic tubules distinguished by dark eosinophilia were also observed in some areas, and atrophic tubules were also observed in some areas. It was observed that the edema in the interstitial areas was significantly less in this group compared to group V. The scoring table for the groups is shown in Table 3 and the observed findings are shown in Figure 2.

**Table 3:** Interstitial edema, edematous tubules, eosinophilic tubules and atrophic tubules count measurement results of the experimental groups.

	C	M	V	V + M	*p*
Interstitial edema	0 (0–1)^a^	0 (0–1)^a^	2 (0–3)^b^	1 (0–3)^c^	0.0001
Number of edematous tubules	0 (0–1)^a^	0 (0–1)^a^	0.5 (0–3)^b^	1 (0–2)^b^	0.0001
Number of eosinophilic tubules	0 (0–1)^a^	0 (0–1)^a^	1 (0–3)^b^	0.5 (0–3)^c^	0.0001
Number of atrophic tubules	0 (0–1)^a^	0 (0–1)^a^	0.5 (0–6)^b^	0 (0–2)^b^	0.0001

*Note:* Data are expressed as median (minimum‐maximum). No statistical difference was found between groups expressing the same letter (a, b, c). *p* < *0.05* was accepted as significant.

Abbreviations: C; control, M; melatonin, V; varicocele, V + M; varicocele + melatonin.

### 3.4 JTBS and Seminiferous Tubule Measurement Results

JTBS and seminiferous tubule diameter, seminiferous epithelial thickness, seminiferous tubule lumen width were calculated. The JTBS score decreased in the V group compared to the C and M groups and increased statistically significantly in the V + M group compared to the V group in Table 5. It was observed that seminiferous tubule diameter, seminiferous epithelial thickness, and seminiferous tubule lumen width were similar in groups C and M. Compared to groups C and M, seminiferous tubule diameter, seminiferous epithelial thickness, seminiferous tubule lumen width and JTBS score decreased statistically significantly in group V. Seminiferous tubule diameter measurements in the V + M group were similar to the C and M groups, but different from the V group. Seminiferous epithelial thickness and seminiferous tubule lumen width increased statistically significantly in the V + M group compared to the V group. The statistical analysis results of these parameters for the experimental groups are shown in Table 4.

**Table 4:** Seminiferous tubule diameter, seminiferous epithelial thickness, seminiferous tubule lumen width and JTBS measurement results of the experimental groups.

	C	M	V	V + M	*p*
JTBS score	6.36 ± 2.1^a^	6.01 ± 1.3^a^	4.05 ± 1.9^b^	5.31 ± 1.5^c^	0.0001
Seminiferous tubule diameter	232.4 ± 46.2^a^	232.9 ± 43.2^a^	210.5 ± 47.9^b^	229.05 ± 39.9^ac^	0.0001
Seminiferous epithelial thickness	46.8 ± 11.8^a^	44.2 ± 8.6^ad^	30.22 ± 16.7^b^	42.2 ± 10.8 ^cd^	0.0001
Seminiferous tubule lumen width	130.2 ± 40.7^a^	134.5 ± 36.1^a^	110.8 ± 34.4^b^	118.7 ± 30.6^b^	0.0001

*Note:* Data are expressed as mean ± standard deviation. No statistical difference was found between groups expressing the same letter (a, b, c, d). *p* < *0.05* was accepted as significant.

Abbreviations: C; control, M; melatonin, V; varicocele, V + M; varicocele + melatonin.

### 3.5 GADD153, GADD45g and GADD34 Immunoreactivity Results

**Figure 3:**
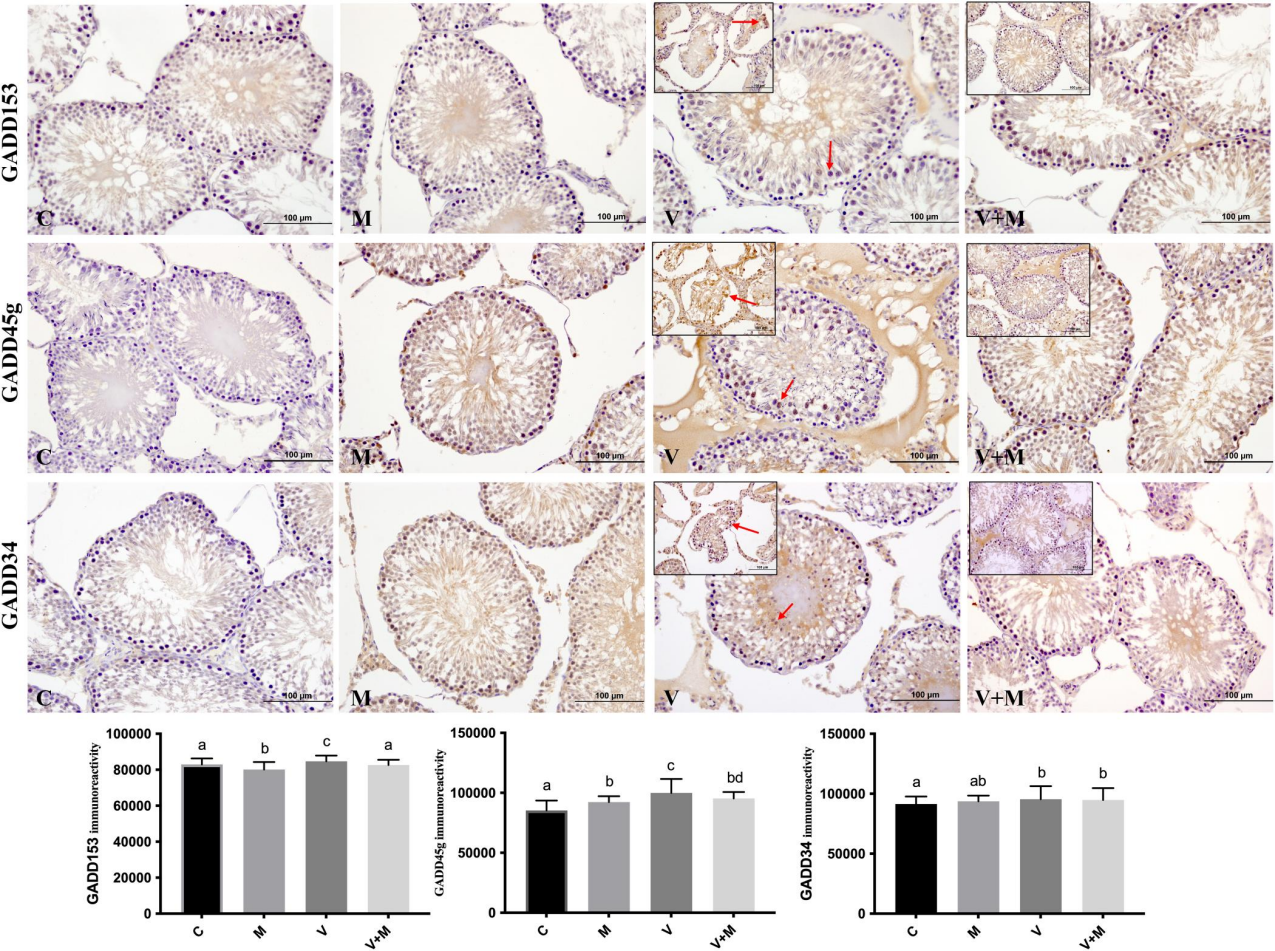
Immunohistochemistry staining results and graphs of the experimental groups' GADD153, GADD45g and GADD34. Expressions are shown with red arrows. Images 40x objective (scale bar: 100 µM). C; control, M; melatonin, V; varicocele, V + M; varicocele+melatonin.

All proteins were expressed primarily in the seminiferous epithelium, as well as in spermatogonia and spermatocytes, and also in interstitial connective tissue. Expression measurement results are shown in graphics at Figure 3. GADD153 expression decreased statistically significantly in the V + M group compared to the V group. It was determined that this decrease was similar to the C group (Figure 3). There was a statistically significant increase in GADD153 expression in the V group compared to the C and M groups. GADD45g expression increased in group V compared to groups C and M. It was determined that there was a statistically significant decrease in GADD45g expression in the V + M group. (Figure 3). The most significant increase due to varicocele was observed in GADD45g expression. GADD34 expression increased in group V compared to groups C and M. This increase decreased slightly in the V + M group (Figure 3).

### 3.6 GDNF and StAR Immunohistochemistry and Gene Expression Results

**Figure 4:**
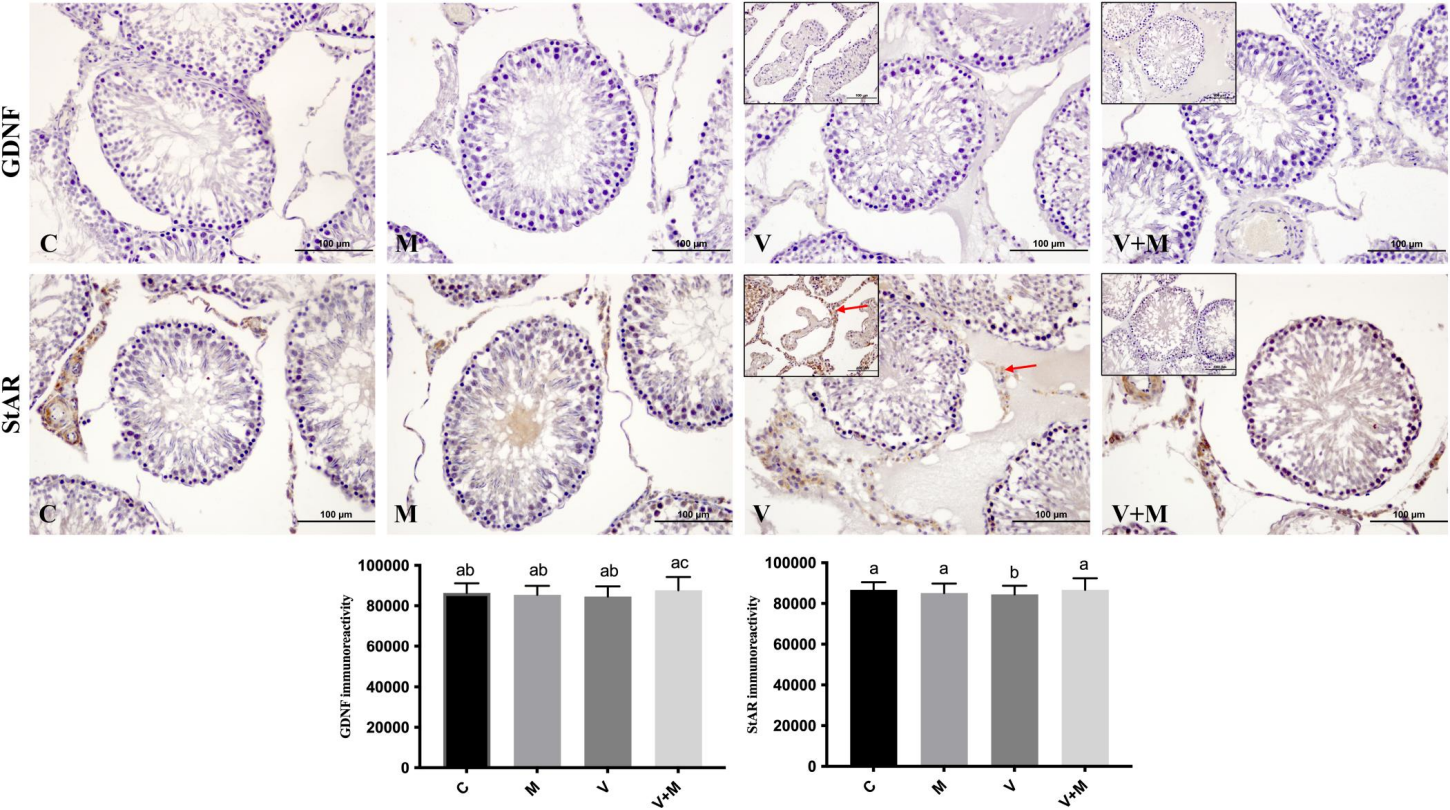
GDNF and StAR immunohistochemistry staining and graphs of experimental groups. Expression intensities are shown with red arrows. Images 40 × objective (scale bar: 100 µM). C; control, M; melatonin, V; varicocele, V + M; varicocele+melatonin.

GDNF gene expression was determined to be decreased in the V group compared to the C and M groups. It was determined that GDNF gene expression increased in the V + M group compared to the V group, and this increase was not statistically significant. Gene expression results of StAR and GDNF are shown in Table 5. StAR immunoreactivity was decreased in group V compared to groups C and M. The V + M group was similar to groups C and M. Although GDNF immunoreactivity was partially reduced in group V, this was not statistically significant. An increase was observed in the V + M group compared to groups M and V. It was determined that StAR gene expression decreased in the V group compared to groups C and M. This decrease was also observed in the V + M group. In addition, immunohistochemistry results of GDNF and StAR are shown in Figure 4.

**Table 5:** GDNF and StAR gene expression analysis results of the experimental groups.

	C	M	V	V + M	*p*
GDNF gene expression	1.73 ± 2.06	1.83 ± 1.2	1.40 ± 0.8	1.74 ± 0.9	0.9291
StAR gene expression	1.33 ± 1.2	1.49 ± 1.1	1.23 ± 0.7	0.89 ± 0.4	0.5870

*Note:* Data are expressed as mean ± standard deviation. No difference was found in gene expression between groups. *p* < *0.05* was accepted as significant.

Abbreviations: C; control, M; melatonin, V; varicocele, V + M; varicocele+melatonin.

### 3.7 Apoptotic Findings

**Figure 5:**
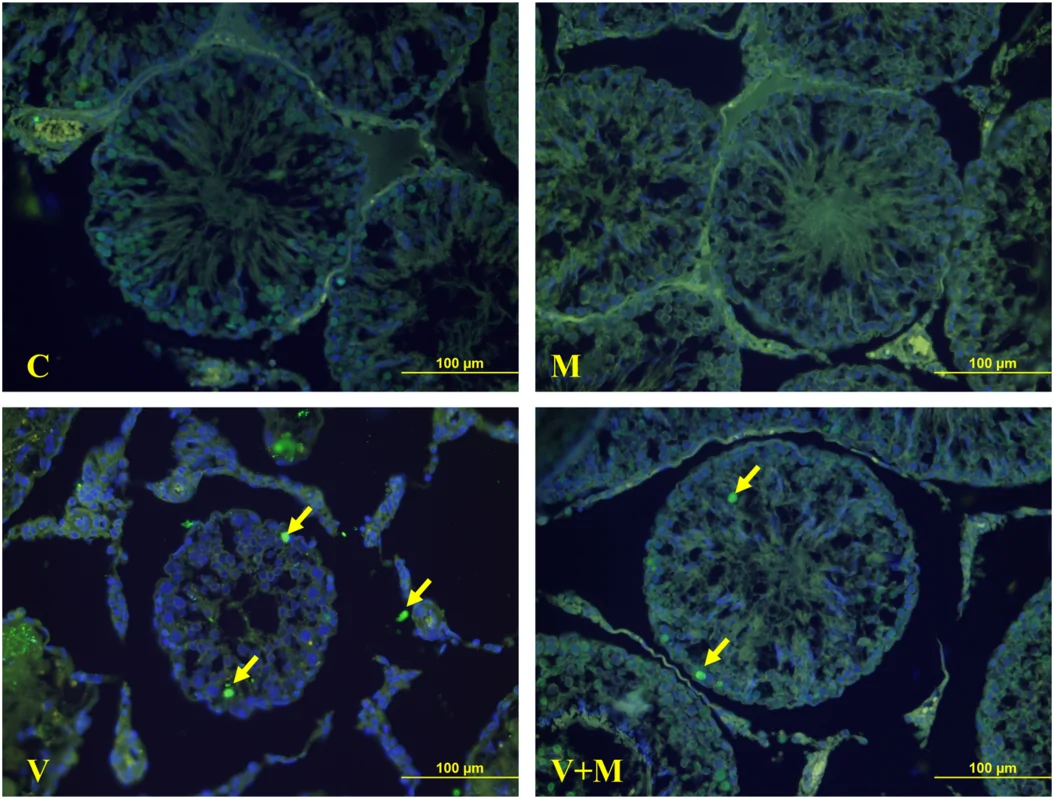
TUNEL‐positive cells (yellow arrows) belonging to the experimental groups are shown. Images were given as 40x objective (scale bar: 100 µm), FITC + DAPI. C; control, M; melatonin, V; varicocele, V + M; varicocele+melatonin, FITC; Fluorescein‐5‐isothiocyanate, DAPI; 4’,6‐Diamidino‐2‐phenylindole4’,6‐ dihydrochloride.

TUNEL positive cells in testicular tissue were observed in seminiferous tubules. Kruskal Wallis test was applied because the groups did not show normal distribution. According to the results, a statistically significant increase was observed in group V. Group V was similar to groups C and M. The results of TUNEL positive cells are shown in Table 6, and the pictures are shown in Figure 5.

**Table 6:** Number of TUNEL‐positive cells in experimental groups.

	C	M	V	V + M	*p*
TUNEL‐positive cells	0 (0–3)^a^	0 (0–3)^a^	0 (0–7)^b^	0 (0–4)^a^	0.0001

*Note:* Data are expressed as mean ± standard deviation. No statistical difference was found between groups expressing the same letter (a, b). *p* < *0.05* was accepted as significant.

Abbreviations: C; control, M; melatonin, V; varicocele, V + M; varicocele+melatonin.

### 3.8 Evaluation of Hormones

Although changes were determined in the hormone levels of the experimental groups, it was observed that there was no statistically significant change in the testosterone, FSH and LH levels. Testosterone decreased in the V group compared to the C and M groups. Testosterone levels increased in the V + M group. FSH decreased in the V group compared to the C and M groups, and it was observed that it increased in the V + M group compared to the V group. LH increased in the V group compared to the C and M groups, and it was observed that the LH level decreased in the V + M group compared to the V group. Tissue ABP levels decreased in the V group compared to the C and M groups. It was observed that there was an increase in the ABP level in the V + M group compared to the V group. The analyses of all hormone levels of the experimental groups are shown in Table 7.

**Table 7:** Analysis of hormone levels in experimental groups.

	C	M	V	V + M	*p*
Testosterone level (blood) (pg/mL)	306.1 ± 138	303.3 ± 11	236.9 ± 105.2	285.7 ± 80.6	0.6241
FSH level (blood) (IU/L)	1.22 ± 0.26	1.26 ± 0.28	1.16 ± 0.23	1.19 ± 0.30	0.7016
LH level (blood) (mlU/mL)	7.25 ± 0.6	7.28 ± 1.3	9.05 ± 1.2	8.93 ± 2.4	0.0381
ABP level (blood) (ng/mL)	12.1 ± 5.3	11.02 ± 3.4	9.79 ± 3.7	13.77 ± 2.3	0.2445

*Note:* Data are expressed as mean ± standard deviation. There is no statistical difference between the groups. *p* < *0.05* was accepted as significant.

Abbreviations: C; control, M; melatonin, V; varicocele, V + M; varicocele+melatonin.

### 3.9 Evaluation of Total Oxidant/Antioxidant Levels

According to our results, TOS levels increased in the V group, while they decreased slightly in the V + M group. At the TAS level, a decrease in the V group relative to the C and M groups was observed, with a slight increase in the V + M group relative to the V group. The analysis results for the groups on TOS and TAS levels are shown in Table 8.

**Table 8:** Analysis of total oxidant/antioxidant levels of experimental groups.

	C	M	V	V + M	*p*
TOS level (tissue) (µmol)	0.54 ± 0.25	0.56 ± 0.25	0.59 ± 0.28	0.57 ± 0.35	0.0062
TAS level (tissue) (mmol)	1.22 ± 0.26	1.26 ± 0.28	1.16 ± 0.23	1.19 ± 0.30	0.0001

*Note:* Data are expressed as mean ± standard deviation. There is no statistical difference between the groups. *p* < *0.05* was accepted as significant.

Abbreviations: C; control, M; melatonin, V; varicocele, V + M; varicocele+melatonin.

### 3.10 Evaluation of Blood and Tissue Melatonin Levels

Since the possible effects of melatonin on varicocele‐induced damage were evaluated in our study, both tissue and blood serum melatonin levels were analyzed. It was observed that both tissue and blood melatonin levels decreased in the V group. In the V + M group, a slight increase in tissue melatonin levels was observed, while an increase in blood melatonin levels was found to be similar to the C and M groups. The analysis results of the experimental groups regarding tissue and blood melatonin levels are shown in Table 9.

**Table 9:** Analysis of tissue and blood melatonin levels of experimental groups.

	C	M	V	V + M	*p*
Melatonin level (Tissue) (ng/L)	25.27 ± 11.7	23.16 ± 7.2	20.51 ± 9.8	20.74 ± 8.08	0.6599
Melatonin level (Blood) (ng/L)	14.1 ± 4.9	14.99 ± 6.3	11.72 ± 3.5	15.7 ± 7.1	0.5536

*Note:* Data are expressed as mean ± standard deviation. There is no statistical difference between the groups. *p* < *0.05* was accepted as significant.

Abbreviations: C; control, M; melatonin, V; varicocele, V + M; varicocele+melatonin.

## 4 Discussion

This article is designed to investigate the pathogenesis of varicocele via ER‐stress in rats who are assumed to have a low endogenous antioxidant system due to varicocele and to reveal whether the change in endogenous melatonin level predisposes to diseases such as varicocele. According to our results, the antioxidant system plays a role in the pathogenesis of varicocele by effectively repairing testicular damage and preserving structural and functional integrity.

Increased intratesticular temperature due to increased pressure in the spermatic vein after varicocele causes disruption of normal spermatogenesis, mass loss and epithelial shedding in atrophied testes, and decrease in sperm concentration and motile sperm count, resulting in a decrease in testicular mass in adolescents and adults with varicocele [17, 18, 19]. We observed that oxidative stress increased due to increased scrotal temperature after prolonged venous reflux in the varicocele testis, which caused dysfunction in both non‐spermatogenic and spermatogenic cell groups, decreased sperm count and motility, and decreased testicular mass. In particular, the appearance of eosinophilic and atrophied tubular structures in the testicular structure, as well as changes in germinal epithelial thickness, reductions in tubule and lumen diameters, and intense edema, affect spermatogenesis, thereby paving the way for infertility. When evaluating both tubular and interstitial edema, it is also necessary to assess whether there is aquaporin (AQP)‐related dysfunction, which is essential for maintaining fluid dynamics and microenvironmental stability in the seminiferous tubules [20]. It should be remembered that this condition can also contribute to testicular damage due to varicocele. It has been suggested that varicocele in the testes can cause irregular tubular shedding, particularly of haploid germ cells, tissue hyperhydration, spermatogenesis failure, and downregulation of AQP‐9 leading to lactate deficiency, potentially disrupting the transport of lactate and water to the germ cells [21]. Therefore, AQPs’ contribution to cell dynamics by mediating the transport of water and dissolved substances in cells, blood vessels, and the interstitial space may underlie varicocele‐related edema [22]. Therefore, both tubular and interstitial edema in the tissue may have resulted from varicocele‐induced AQP downregulation. Explaining the cause of the edema levels observed in the V + M group, depending on the experiment duration and melatonin administration dose, does not seem possible because AQP levels were not directly analyzed. This is a limitation of this article. The presence of eosinophilic tubules also indicates necrosis. It is known that semen quality gradually decreases with the severity of varicocele [6]. Therefore, we can think that the reason why fertility among people suffering from varicocele varies from individual to individual is related to the severity of the varicocele. Varicocele‐induced testicular hyperthermia triggers various adaptive responses in the organ, such as ER‐stress [23]. ER‐stress activates a series of adaptive mechanisms known as the unfolded protein response. ER dysfunction is a potent inducer of the transcription factor CCAAT/enhancer‐binding protein (C/EBP) homologous protein, CHOP [24]. CHOP, also known as growth arrest and DNA damage‐inducible gene (GADD153), plays a role in regulating processes related to cellular proliferation, differentiation, and expression. While GADD153 expression is normally extremely low in the cell, it increases during ER‐stress‐induced apoptosis [25]. Genes that include GADD153 as well as GADD45 and GADD34 are a group of genes induced by genotoxic stress and growth arrest signals. There is no similarity between these GADD genes [26]. The GADD45 gene family consists of small evolutionarily conserved proteins called GADD45a, GADD45b, and Gadd45g. They act as stress sensors in response to physiological and environmental stress [27]. Our study aimed to demonstrate GADD45g expression, which plays an important role in gender differentiation and is essential for male infertility and testicular development [28]. GADD34 expression is also induced in cellular stress conditions such as DNA damage and ER‐stress [29]. GADD153 is produced during ER‐stress and is expressed during ER dysfunction, allowing only correctly folded proteins to be sequestered, so its expression is low in normal cells and participates in ER‐stress‐induced apoptosis [30]. In our study, GADD153 expression increased less than GADD34 and GADD45g. If we consider the eosinophilic‐stained seminiferous tubules in group V as an indicator of possible necrosis mechanisms in the tissue and assume that GADD153 also increases in apoptosis, it may explain why GADD153 increases less than GADD45g and GADD34. In our study, we found that GADD45g was more highly expressed in varicocele testes than GADD153 and GADD34. This is due to the involvement of GADD45g in male infertility as mentioned above. Because the expression of these genes is often increased in response to stressful environments, which may result in cell cycle arrest and/or cell death [31]. GADD45g is involved in various responses to cell damage, including DNA replication and repair in the cell cycle [32]. Increased GADD34 expression prevents the inhibition of protein synthesis in the cell and plays an important role in autophagy [33]. Given that these genes are coordinately expressed and induced by certain growth arrest genes, we can explain why GADD45g and GADD34 are increased in the testicular germinal epithelium undergoing continuous renewal. We believe that the expression of these genes increases to prevent cell death from oxidative stress caused by varicocele. We used TUNEL to assess apoptosis and found that varicocele increased apoptosis. The increase in GADD153 levels due to ER stress can indicate that apoptosis is triggered in the cell. Here, melatonin reduces the expression of all three GADD proteins, suggesting that it contributes to the inhibition of ER stress. Because melatonin acts as a powerful free radical scavenger. In this study, the evaluation of both spermatogenic and non‐spermatogenic cells together demonstrates the effect of melatonin. Sertoli cells in the germinal epithelium produce glial cell‐derived neurotrophic factor (GDNF) to maintain the self‐renewal of spermatogonial stem cells. GDNF is important for spermatogonial cells to form spermatocytes [34]. Leydig cells play a critical role in male reproductive physiology, and dysfunction in these cells is associated with infertility. Steroidogenic acute regulatory protein is a mitochondrial protein whose release is induced by luteinizing hormone (LH) and is necessary for the cell to produce steroids [35]. Therefore, StAR is a critical protein for Leydig cell function. Studies have also reported that the decrease in GDNF and StAR mRNA levels causes the disruption of the Sertoli‐Leydig cell network function [36, 37]. Therefore, in evaluating the relationship between Leydig cells and Sertoli cells, the presence of testosterone and ABP was also taken into account. Our study revealed that varicocele decreased StAR and GDNF mRNA expression. These results can be considered an indication that damage to the seminiferous tubules and interstitial connective tissue is significant. According to our results, the presence of melatonin does not cause a significant change in StAR expression, while it is more effective on GDNF. These results suggest that melatonin may be more effective in the seminiferous tubule structure rather than Leydig cells. The changes in hormone levels examined in our study are important in terms of showing the cell function expressed by these genes. Spermatogenesis is a highly regulated process and is controlled by hormones, particularly testosterone and the gonadotropins FSH and LH. In our study, it was observed that varicocele caused a decrease in testosterone, FSH and ABP levels and an increase in LH levels. Testosterone is a key hormone for the spermatogenesis process and is necessary for the initiation and maintenance of spermatogenesis. LH is secreted from the pituitary gland and acts on the Leydig cells, stimulating them to secrete testosterone [38]. The LH level may have increased to correct the decreased testosterone level due to impaired Leydig cell function after varicocele. Because varicocele has been reported to disrupt serum testosterone, FSH and LH levels through its effects on Leydig cells, Sertoli cells and the hypothalamic‐pituitary‐gonadal axis [39]. In addition to its direct effects on spermatozoa, varicocele also has adverse effects on other testicular cell types. Clinically, it is associated with Sertoli cell dysfunction, decreased response to FSH, and changes in ABP, transferrin, and inhibin. It has been reported that varicocele occurs in some men with increased FSH and low testosterone production [40]. In our study, the decrease in testosterone and ABP after varicocele is another important piece of evidence for demonstrating the Sertoli‐Leydig dysfunction that we targeted. This decrease is parallel to the decrease in both mRNA and protein expression of GDNF and StAR. Melatonin has high lipid and water solubility, which facilitates its passage through cell membranes, allowing it to easily pass into the cell and scavenge toxic hydroxyl radicals and other oxygen‐centered radicals [41]. Therefore, it reduced the varicocele‐induced TOS level and caused improvement in the TAS level. It should be noted that melatonin production is secreted less in the first years of life, reaches its highest level at the age of 1‐3, and gradually decreases in adulthood [7]. Considering that varicocele is a disease that begins to appear at an early age and its incidence increases with age, and that advancing age causes a gradual decrease in melatonin levels, it is inevitable that the relationship between varicocele and antioxidants should be revealed. There are no studies showing how blood melatonin levels change after a varicocele. Therefore, we also evaluated the relationship between varicocele and melatonin levels. We observed changes in both tissue and blood melatonin levels after varicocele. The increases in blood and tissue melatonin levels were not parallel. This was probably due to the time it takes for melatonin to affect the tissue. If we had applied exogenous melatonin for more than 7 days, our results in all parameters would probably have been more significant. While the 10 mg/kg melatonin dose we used in this study may be considered safe in rat studies, it is important to remember that it differs markedly from the recommended dose for human use in supplements. In this study, using this dosage, we investigated the mechanistic/genetic pathways underlying varicocele‐induced testicular damage, and we hope that the data we obtained will serve as a reference for future studies involving human use.

## 5 Conclusion

Since effective results are obtained in the presence of melatonin by reducing testicular damage developing after varicocele and regulating the oxidant/antioxidant system and hormone levels, it can be said that the presence of exogenous melatonin has an important place in reducing varicocele‐induced damage. Another important result here was that increased GADD expressions after ER stress decreased in the presence of melatonin. In addition, when spermatogenic cell line and non‐spermatogenic cell function were evaluated via GDNF and StAR, it was revealed that melatonin may have a regulatory effect on both Sertoli and seminiferous epithelium and Leydig cells. Although this study has shown that an antioxidant molecule used endogenously in the body can contribute to the infrastructure of various diseases, studies are needed to increase the duration of melatonin application to obtain statistically more effective results.

## Author Contributions

The authors declare that all data were generated in‐house, that no paper mill was used and that no AI tool has been used for the generation of text or figures. Conceptualization: Esra Önal, Numan Baydilli, Derya Karabulut. Data curation: Esra Önal, Derya Karabulut. Formal analysis: Esra Önal, Numan Baydilli, Derya Karabulut. Methodology: Esra Önal, Numan Baydilli, Derya Karabulut. Project administration: Numan Baydilli, Derya Karabulut. Visualization: Esra Önal, Derya Karabulut. Writing – original draft: Derya Karabulut.

## Conflicts of Interest

The authors declare no conflicts of interest.

## Data Availability

Data will be made available on request.
